# Hiding in Plain Sight: Pulmonary Vein Stenosis Following Pediatric Heart Transplantation

**DOI:** 10.1111/petr.70199

**Published:** 2025-10-09

**Authors:** Conor P. O'Halloran, Amanda Hauck, Anna Joong, Paul Tannous

**Affiliations:** ^1^ Ann & Robert H. Lurie Children's Hospital of Chicago Chicago Illinois USA; ^2^ Northwestern University Feinberg School of Medicine Chicago Illinois USA

## Abstract

**Background:**

Pulmonary vein stenosis (PVS) after pediatric heart transplantation (PHT) is an observed phenomenon with previously unknown incidence, risk factors, treatment, and outcome.

**Methods:**

This is a review of three recent publications describing PVS after PHT.

**Results:**

In total, 712 PHT recipients from four centers, over a combined 43 years, are reviewed. Thirty‐one new cases of PVS after PHT, in addition to six patients with preexisting PVS, are described. PVS diagnosis occurred in the first year after PHT for most patients. Left‐sided PVS were more than twice as common as right‐sided PVS. Nearly half (43%) experienced multivessel PVS. Major risk factors of PVS after PHT included younger age, history of congenital heart disease (CHD), and history of anomalous pulmonary venous return. The treatment of PVS after PHT varied, reflecting uncertainty in the management of PVS generally. With a median follow‐up of less than 3 years, 19% of patients with PVS after PHT died.

**Conclusions:**

PVS after PHT complicated approximately 4.4% of cases in these reports. PVS is more common after PHT in younger patients with a history of CHD. PVS is generally diagnosed in the first year after PHT. We recommend careful evaluation for PVS in the first year after PHT in patients with known risk factors.

**Abbreviations**
BSA: body surface area
CHD: congenital heart disease
PHT: pediatric heart transplantation
PVS: pulmonary vein stenosis

## 1 Introduction

Pulmonary vein stenosis (PVS) is a rare disease characterized by progressive neointimal proliferation [1, 2, 3]. This can result in pulmonary hypertension, respiratory failure, right ventricle dysfunction, and death. Recent therapies have improved the outcome for this disease, but morbidity and mortality remain high. The management of PVS varies significantly, with several pediatric cardiac centers developing comprehensive care teams to address this condition. Although the occurrence of PVS following pediatric heart transplantation (PHT) has been observed clinically, it has not been systematically characterized until recently.

PHT is the standard of care for the treatment of end‐stage heart failure in children, most often due to congenital heart disease (CHD) or cardiomyopathy. Advances in perioperative, intensive care, and immunosuppression management have led to improved median graft survival, which in infants currently exceeds 24 years [4]. Morbidity remains high, and PHT recipients are at risk for numerous complications including rejection, infection, coronary allograft vasculopathy, and malignancy [5].

In the past year, three independent studies have described the occurrence of PVS after PHT across four large‐volume centers, spanning a combined observation period of more than 40 years and 700 PHTs [6, 7, 8]. In this work, we review these three papers, with a focus on findings common to all studies (see Table 1 for raw and summative data). The goal of this commentary is to create a coherent framework for understanding this specific disease process to guide clinical management and future research.

**TABLE 1:** Summary of published data.

	Combined *N* = 37	Choi et al. *N* = 6	Butto et al. *N* = 19	Takajo et al. *N* = 12
Observation period (years)	43 center‐years	10	11.5 (2 centers)	10
Heart transplant recipients (total)	712	176	422	114
De novo PVS diagnoses	31 (4.4%)	6	15	10
Pre‐transplant‐PVS	6 (0.8%)	Excluded	4	2
Congenital heart disease	34/37 (92%)	6/6	16/19	12/12
Age at transplantation (months)		7.5 (range 2–13)	23 (IQR 8–42)	24 (IQR 4.8–60)
Time to diagnosis (months)		3.5 (range 0.3–13)	2 (range 1–168)	2 (IQR 1–8)
Method of PVS diagnosis				
Echocardiography	21	3	8	10
Catheterization	16	3	11	2
CT/MRI	6	0	6	0
Specific veins involved				
Left lower	18	4	11	3
Left upper	14	2	5	7
Left common	2	1	1	0
Right lower	7	1	2	4
Right upper	5		4	1
Multivessel disease				
At diagnosis		1	6	(not reported)
At follow up	16/37 (43%)	2	11	3
Treatment				
Any structural therapy	25/33 (76%)	6	13^a^	6
Stent placement		5	6	(not reported)
Surgical repair		0	4	0
Median follow up (months)		27	35	29
Mortality	7/37 (19%)	0	3	4

Abbreviations: CT = computed tomography, IQR = inter‐quartile range, MRI = magnetic resonance imaging, PVS = pulmonary vein stenosis.

^a^ 13/15 patients with de novo PVS underwent intervention.

## 2 Pulmonary Vein Stenosis

PVS can be categorized as primary PVS that occurs in the absence of prior pulmonary venous surgery or secondary (post‐repair) PVS if it occurs following surgical intervention of anomalous pulmonary venous return. Neointimal myofibroblast and extracellular matrix proliferation is the final common pathway in progressive disease resulting in loss of vessel area, pulmonary hypertension, and in severe cases RV failure and death [3]. Several clinical risk factors and comorbidities are associated with the development of PVS including prematurity and bronchopulmonary dysplasia, infection/inflammation, aspiration, left‐to‐right congenital cardiac shunts, and genetic syndromes [9, 10, 11, 12].

Multiple inciting anatomic mechanisms have been proposed to contribute to the development of PVS including external compression by the spine, descending aorta, or bronchi, as well as increased sheer stress from an abnormal angle of pulmonary vein insertion to the left atrium [13]. Treatment approaches vary by center, but generally include both medical therapies and structural intervention. With earlier identification and aggressive comprehensive management, outcomes have improved in recent years [14, 15].

## 3 PVS in PHT: Incidence

Two of the three recent studies are single‐center reviews of 10 years of PHT recipients, while the other study reviews 11.5 years at two transplant centers. Collectively, these studies include 712 PHT recipients. Preexisting PVS was an exclusion criterion for one study, while the other two studies included a total of six patients with a preexisting diagnosis of PVS. Therefore, the total reported cohort of PHT recipients without preexisting PVS (i.e., the number at risk for de novo PVS) was 706. The follow‐up period from transplantation was variable, as all studies were retrospective reviews and only one study explicitly states the median follow‐up period for the transplant population at large. Excluding the six patients with preexisting PVS, 31 new cases of PVS were observed; thus, the observed incidence of de novo PVS after PHT was 4.4%. Certainly, the true incidence of post‐PHT PVS may be different from this reported value due to missed diagnoses or publication bias.

The diagnosis of PVS generally occurred within the first year after PHT. The median time to diagnosis in the three studies was 3.5 months, 2 months, and 2 months. One patient was diagnosed 14 years after PHT, which suggests that not all cases of PVS after PHT receive timely diagnosis, and some may be missed altogether. PVS was diagnosed most frequently by echocardiogram, followed by cardiac catheterization. Multiple centers confirmed an echocardiographic diagnosis with CT angiography. The specific veins affected are reported in all three studies. The most affected vein was the left lower pulmonary vein, which was stenotic in 22/37 patients. Left‐sided vein stenosis was more common than right‐sided vein stenosis (34 left‐sided vs. 15 right‐sided veins). Multivessel disease was present in 16/37 (43%) of patients, including those with preexisting PVS. In the two papers in which this data is presented, 6/18 (33%) patients initially diagnosed with single vessel PVS progressed to multivessel disease during the study observation period.

## 4 PVS In PHT: Patient Characteristics and Risk Factors

All three papers describe the demographics of the patients with PVS after PHT. In general, these patients were young, with a median age at the time of transplant ranging from 7 to 24 months. More than 90% of patients received PHT for a history of CHD. Comparisons between PHT patients who did and did not develop PVS found that younger age, history of CHD, history of partial anomalous pulmonary venous return (PAPVR), and presence of systemic venous obstruction were all associated with post‐PHT PVS. Notably, sex, race, use of ventricular assist device, and donor‐to‐recipient size ratios were not associated with post‐PHT PVS. In the one paper that reported on the diagnosis of heterotaxy, it was present in 17% of those with PVS compared to 4.5% of those without PVS, but this was not significant (*p* = 0.06). One paper examined infection and rejection and found 6/6 PVS patients experienced infection while 0/6 experienced rejection before the diagnosis of PVS was made.

## 5 PVS in PHT: Treatment and Outcome

The treatment of PVS after PHT was quite variable. Only 8/37 patients did not undergo any structural intervention (i.e., catheter‐based therapy or surgery). The remaining patients had balloon angioplasty, stent placement, or surgery. The relative use of these therapies differed by center, reflecting the variability in treatment approaches for PVS in general. The median follow‐up time from PVS diagnosis was 28, 29, and 35 months in the three studies. Including those with preexisting PVS, 7/37 (19%) patients died. Only one study collected mortality data for the entire PHT cohort to be able to compare mortality to those with and within PVS; in this study, they found that PVS was associated with a higher risk of mortality (*p* = 0.025) using the Breslow test, though the log‐rank test was not significant (*p* = 0.063).

## 6 Conclusions

**FIGURE 1:**
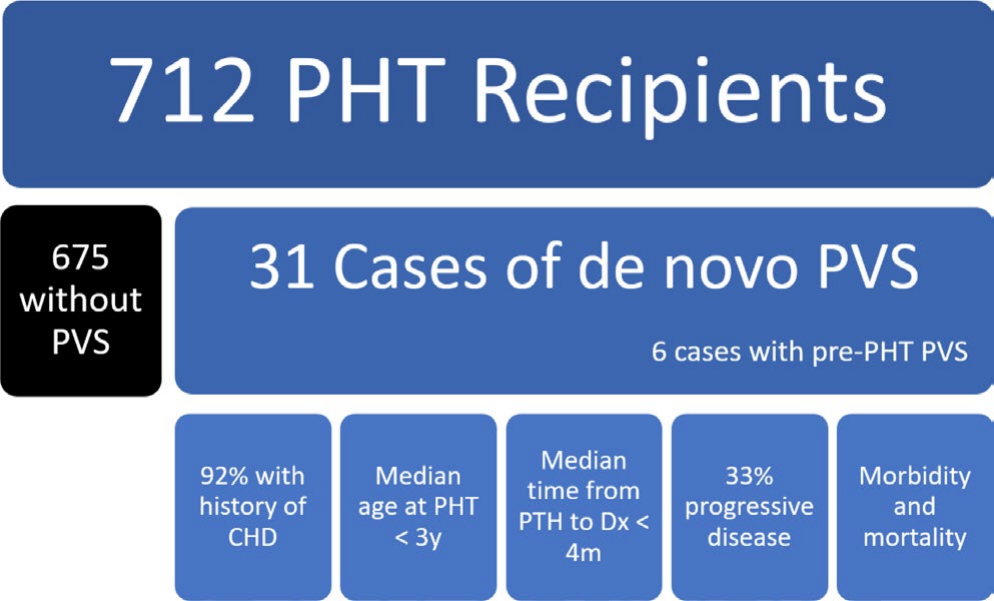
Infographic demonstrating the collective findings from published works discussing pulmonary vein stenosis after pediatric heart transplantation. CHD = congenital heart disease, Dx = diagnosis, PHT = pediatric heart transplant, PVS = pulmonary vein stenosis.

In conclusion, de novo PVS complicates approximately 4.4% of PHTs, with the vast majority of cases diagnosed in the first year following transplant, in patients with a history of CHD, and younger age at the time of PHT (Figure 1). For comparison, this exceeds the first‐year rates of malignancy (1%), PTLD (1%), and cardiac allograft vasculopathy (3%) [4, 5, 16]. PVS appears to be significantly more frequent following PHT for CHD compared to cardiomyopathy. A history of anomalous pulmonary vein repair appears to be an additional risk factor, but most patients with PVS after PHT did not have anomalous veins; thus, surveillance should not be limited to this group. Treatment of PVS after PHT is extremely variable, reflecting the lack of consensus in the management of PVS generally. Patients who develop PVS after PHT appear to have reduced survival compared to non‐PVS, though more data are needed. The non‐transplant PVS literature suggests early aggressive intervention can improve outcomes. Thus, we suggest that dedicated surveillance for PVS after PHT is crucial, especially during the first year posttransplant in young patients with a history of CHD.

## Ethics Statement

Institution review board approval was obtained with waiver of the need for informed consent.

## Conflicts of Interest

The authors declare no conflicts of interest.

## Data Availability

The data that support the findings of this study are available from the corresponding author upon reasonable request.
